# Glycated albumin: an overview of the *In Vitro* models of an *In Vivo* potential disease marker

**DOI:** 10.1186/2251-6581-13-49

**Published:** 2014-04-07

**Authors:** Amir Arasteh, Sara Farahi, Mehran Habibi-Rezaei, Ali Akbar Moosavi-Movahedi

**Affiliations:** 1 Department of Microbiology, Faculty of Science, Rasht Branch, Islamic Azad University, Rasht, Iran; 2 School of Biology, College of Science, University of Tehran, Tehran, Iran; 3 Institute of Biochemistry and Biophysics, University of Tehran, Tehran, Iran

**Keywords:** Glycation, Human serum albumin, Disease marker, Diabetes

## Abstract

Glycation is a general spontaneous process in proteins which has significant impact on their physical and functional properties. These changes in protein properties could be related to several pathological consequences such as cataract, arteriosclerosis and Alzheimer’s disease. Among the proteins, glycation of Human serum albumin (HSA) is of special interest. Human serum albumin is the most abundant protein in the plasma and because of its high sensitivity for glycation, undergoes structural and functional changes due to binding of reducing sugars *in vitro*. The glycation process occurs by plasma glucose *in vivo* which has great impacts on the three dimensional structure of protein. These changes are efficient and stable enough which makes the protein to be considered as a new special disease marker instead of HbA1C for diabetes. In some cases, glycated albumin was used as an alternative marker for glycemic control. Glycated albumin reacts with glucose ten times more rapidly than HbA1C and has shorter half-life which makes it more reliable for indicating glycemic states. In this review, glycation of Human Serum Albumin has been overviewed, starting from overall concepts of glycation, followed by some Examples of pathological consequences of protein glycation. The BSA aggregation was reviewed in terms of structural and biological impacts of glycation on the protein followed by reporting documents which indicate possibility of glycated albumin to be used as specific marker for diabetes. Finally, some of the studies related to the models of glycated albumin have been briefly described, with an emphasis on *In vitro* studies. It is interesting to note the relationship found between *in vitro* glycation experiments and the propensity of proteins to form amyloid structures, a point that could be further explored as to its significance in hyperglycemic states.

## Introduction

Albumin is one of longest known proteins of plasma [1]. Normal concentration of albumin is 35–50 g/l, which makes it the most abundant protein in plasma with a wide variety of physiological functions. Human albumin presents 50% of the normal individual’s plasma protein [2]. The protein is organized into three domains, I, II and III, each subdivided into two subdomains, A and B [3], with 17 intramolecular disulfide bonds which makes it suitable for a wide variety of modifications including response to pH and other biophysical compounds [4]. Due to its low molecular weight (67 kDa), albumin contributes in osmotic pressure maintenance of plasma, compared with other plasma globulins [5], and also because of its weak isoelectric point, the protein has a global negative charge at physiological pH [6]. Albumin structure allows protein to bind and transport diverse metabolites such as metal ions, fatty acids, bilirubin and drugs [7,8]. Indeed, conjugation of drugs with this protein with long half-life, improved their pharmacokinetic properties [9].

There are three main binding sites on the protein; two of them (site I and site II) are located on subdomains IIA and IIIA, respectively [10] and have been found to bind specifically aromatic and heterocyclic ligands [3]. Flexibility of human serum albumin (HSA) enables interaction of the protein with numerous compounds, by contrast, site II, which is less flexible, has not same property. As examples, paracetamol, a commonly used analgesic drug, binds to residues located in the subdomain IIIA [11,12] and other metabolites such as fatty acids bind to other locations, whose seven binding sites are localized in subdomains IB, IIIA, and IIIB [13]. In addition, some residues, such as cysteine, lysine, serine and arginine, have found to covalently bind to many drugs [14].

When protein modification induced by physiological or pathological changes occurs, an alteration of the native conformation and efficiency of these binding sites can be expected [15]. Advanced Glycation End Products (AGE), which could bind to plasma proteins, were considered as a novel class of uremic toxins [16], and besides ceruloplasmin and other plasma compounds such as ascorbic acid [17], the role of albumin has been highlighted as an antioxidant, having a part in keeping the body safe in cases of oxidative stress [18] which is due to availability of several residues to work as antioxidant [19]. Cys-34 is one of the powerful residues involved in disulfide bond and in a healthy person 70-80% of albumin Cys-34 is in its reduced form and is able to scavenge hydroxyl radicals [20] when the protein is in native conformation [21].

Another amino acid, methionine, is involved in the antioxidant activity of protein. The more exposed methionine residues are presented to oxidation, could serve as ROS (reactive oxygen species) scavenging system to protect proteins from oxidation [22]. Other main binding sites could be mentioned. For instance, carbohydrates could bind to albumin via three main sites (Lys-351, Lys-475 and Arg-117), and as so, albumin may protect other proteins from glycation in the initial stages of diabetes [23].

Many studies have been done on bovine serum albumin (BSA) which has high (76%) sequence homology to human serum albumin (HSA). BSA, with 583 amino acids and molecular weight of 66.28 [24], has an ellipsoidal shape and like HSA, includes three homologous domains (I, II and III), which are connected together through helical extensions. Similar to HSA, it contains 17 disulfide bridges which make the structure stable in neutral pH and room temperature. The free thiol group of Cys-34 is also present in BSA. It has been found to have a relevant role in thermal aggregation of the protein [25]. BSA shows several conformational isomers at different pH values; lots of these structural changes are due to breakage of ionic bonds. The three dimensional structure of the protein changes from nature (N) form, hold at pH 4–8, to the unfolded state at pH 3, where a decrease of helical structure is observed [26].

As mentioned, BSA and HSA have close homology in primary structure; a main difference, important in structural studies, is believed to be due to the presence of two tryptophan residues (Trp-131, and Trp-214) in BSA, while one (Trp-214) is present in HSA [27,28]. The tryptophan 214 localized at domain II of HSA is suggested to have a role in its amyloid fibril formation [29].

In this review, we provide an outline on human serum albumin and its glycation in diabetic patients, which is recently suggested to have high potential as a possible diagnostic marker alongside with the well-known HbA1C. Some pathological effects of glycation are also mentioned. Finally, an introduction to *in vitro* models of albumin glycation, as valuable tools in the study of this modified protein is also included.

### Glycation of proteins

Glycation, i.e., the non-enzymatic addition of carbohydrate moieties to protein reactive residues, has been the subject of many studies over the last decade. The process is fastened in diabetic states, which provided the preliminary ground to assess the relationship between elevated levels of glycated hemoglobin A1C and this disease [30]. *In vivo* glycation has been described in proteins such as the lens crystallins [31], collagen, ferritin, apolipoprotein [32], and serum albumin [33]. In addition to glucose, sugars such as galactose [34], fructose, ribose [35] sialic acid [34], mannose [36], glucose 6-phosphate [37], glyceraldehydes [38], and fucose [39] have been used *in vitro* as glycating agents, sometimes to fasten the process.

These sugars undergo maillard reaction corresponding to a condensation between a carbonyl compound of reducing sugar and a free amino group of specific residues such as lysine or arginine [40] and besides the solubility of the end-stage products, the reaction intermediates may be dark brown aggregates [41]. Early glycation leads to the formation of Schiff's bases and Amadori products and Further oxidation produces advanced glycated end products (AGE) [42]. During the production of fluorescent AGEs products, proteins undergo both glycation and oxidation. As the early-stage of the reaction includes interaction of reducing sugars, such as glucose, with free amino groups of lysine and arginine residues, recent studies have demonstrated that the glycation process is facilitated readily by the presence of a histidine residue near the lysine [43]. The late-stage of reaction is an irreversible cascade of reactions involving dehydration, hydrolysis, etc., leading to the formation of AGE. AGEs products are considered to be a marker of various diseases, such as arteriosclerosis, renal failure, Alzheimer disease, or diabetes, but they also increase during normal aging [44,45].

**Figure 1:**
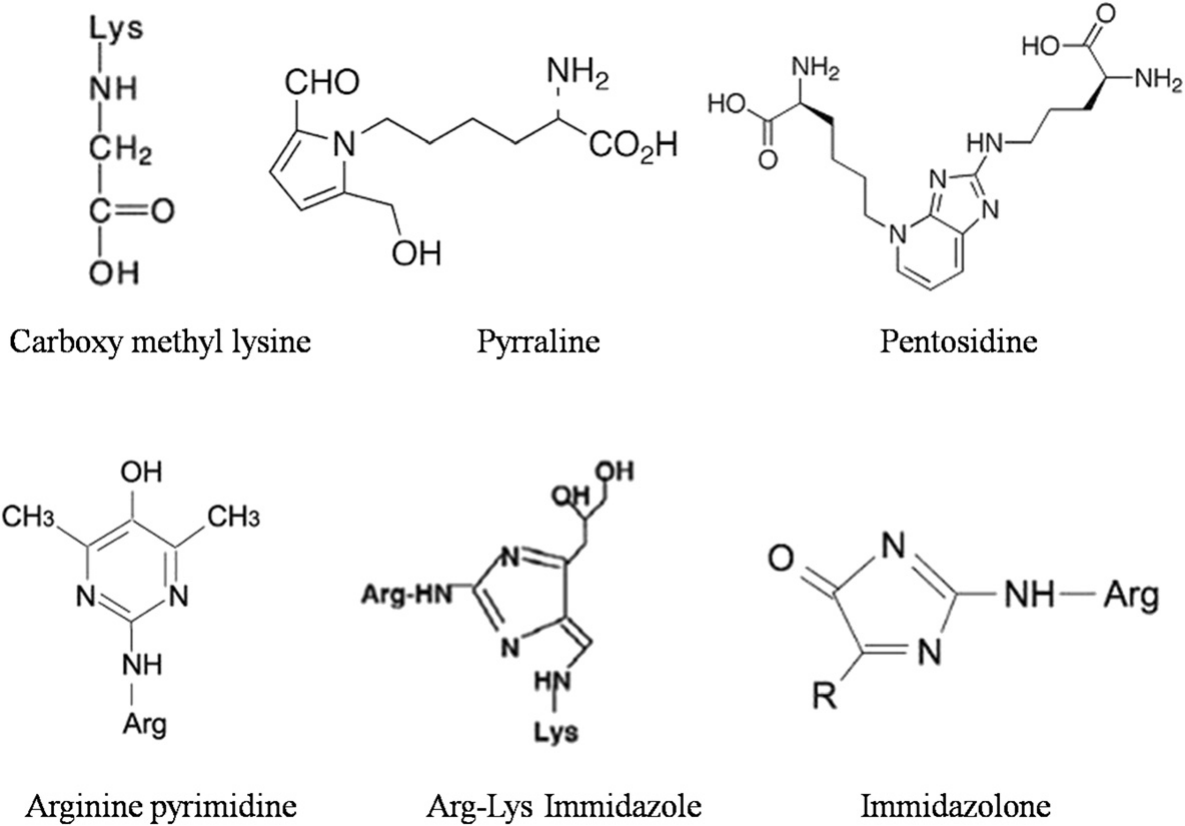
Chemical structures of various AGEs.

AGE products exhibit a wide range of chemical structures and thereby have different biological [46,47] properties (Figure 1). Major chemically characterized AGEs are *N*-(carboxymethyl)lysine (CML) [48], pentosidine [49], pyrraline [50], and imidazolone [51].

Protein cross-linking by AGEs results in the formation of detergent-insoluble aggregates [52,53]. Such aggregates may have interference with protein degeneration and other metabolic disorders during the diabetes or other *in vivo* glycating conditions in the body such as pregnancy.

### Examples of pathological consequences of protein glycation

Most of the studies on glycation contributed to diseases have been primarily focused on diabetes and diabetes-related complications [54,55]. However, resulting damages are, of course, not limited to diabetic patients and even at normal glucose levels some degree of glycation and resulting damage occurs. Such damages are observed in various diseases such as cataract, arteriosclerosis, and neurodegenerative diseases such as Alzheimer’s disease (AD), Parkinson’s disease [56], Creutzfeld-Jacob disease [40] and amyotrophic lateral sclerosis (ALS) [33].

In general, these random damage-induced posttranslational modifications of proteins are basic causatives of protein degradation [57-60]. However, this oxidative cross linking of proteins is accompanied by a decreased proteolytic susceptibility and therefore accumulation of these oxidized proteins [61]. For example Friguet et al. [62] reported a decreased proteolytic susceptibility of N-carboxymethyl lysine- glycated glucose-6-phosphate dehydrogenase. Besides that, the degradation of AGE modified proteins might be a complex process, including various uptake mechanisms, several proteases or peptidases, and perhaps other additional hydrolases, involving in lipid modifications [63].

### Cataract

Cataract is one of the major causes of impaired vision which caused blindness in patients with long time elevated glucose levels. Indeed diabetes has been thought to increase the risk of its development. At least two mechanisms have been mentioned for development of cataract in diabetic subjects: firstly, the aldose reductase-osmotic mechanism and, secondly, the glycation hypothesis. As a matter of fact, the glycation hypothesis may be more compatible with the slow progress of cataract in diabetic patients.

A lot of reports, confirms glycation of lens crystallin in the case of diabetic cataract [64-66]. Liang et al. (1986) reported increased glycation of α-crystaliin compared to β and γ-crystallins. The data of Stevens et al. (1978) showed that glycation of γ-crystallin was higher than α and β-crystallins in diabetic rats and also Chiou et al. (1981) observed that γ -crystallin glycation was equal to or higher than that of α and β-crystallins and other studies showing glycation of different crystallins. In rat lens, the soluble protein fraction diminishes considerably with aging and diabetes and the proportion of different crystallins depleted from the soluble fraction may also vary [64]. It is believed that glycation causes lens crystallins to aggregate into high molecular weight (HMW) aggregates [67].

### Arteriosclerosis

Arteriosclerosis in its wide sense, including athero- and arteriosclerosis occurs more frequently in diabetics than in non diabetic individuals [68]. Diabetes leads to arteriosclerotic diseases such as coronary artery occlusion and cerebrovascular. In addition, hyperglycemia causes aortic intramural accumulation of sorbitol, which leads to increased osmolarity and thus to intramural water retention and decreased tissue oxygenation [69]. Glycation of low density lipoprotein and collagen in the vascular wall are also considered as effective factors of arteriosclerosis [70]. Elastin is another important protein component of elastic fibers of arterial media with a long biological half-life which glycation causes macroangiopathy in diabetic individuals [71]. Actually the elastin content per unit volume of thoracic aorta decreases more rapidly in diabetics than in normal control individuals. Indeed the content of collagen and glycosaminoglycan increases in parallel with calcium deposition and therefore accumulation of degenerated elastin in diabetics.

### Alzheimer’s disease

Alzheimer’s disease (AD), the leading cause of senile dementia, is another glycated protein related disease which is characterized by formation of senile plaques in brain. A wide variety of studies have demonstrated the increased accumulation of AGEs in AD brain [72-74]. AGE modifications and oxidative-stress mechanisms can both impair the neuronal proteins and result in plaques formation [75]. Amount of glycation in cerebrospinal fluid (CSF) is another cause of ageing and AD. Indeed, glycation of CSF proteins which is reported in the CSF of AD patients is suggested to be a triggering cause of AD. It is proposed that the high level of glycation in AD may be accompanied by numerous neuropathological consequences due to damaged proteins [72].

### Glycation of albumin

Human Serum Albumin is very sensitive to glycation. As mentioned before, the slow, non-enzymatic Maillard reaction initially involves the attachment of glucose or other carbohydrate compounds such as galactose and fructose, to the free amine groups of albumin [35]. The glycation efficiency depends on the nature and the anomerization of the carbohydrate involved in the process. As an example, in comparison with glucose, ribose induces a faster glycation process with albumin and forms amyloid-like products [76]. *In vivo* studies demonstrated that the proportion of glycated albumin in healthy persons is in the range of 1- 10% [3], compared with diabetic individuals in whom this may increase two- to three fold [77].

### Impact of glycation on albumin structure

**Figure 2:**
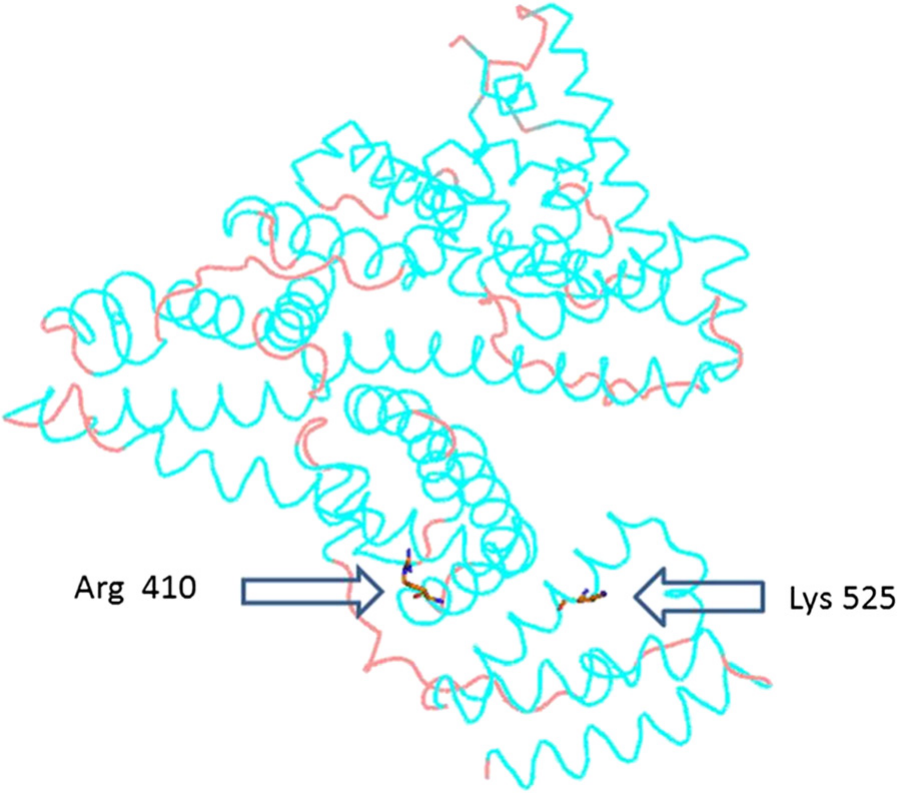
**Human serum albumin drug binding sites with the location of main lysine and arginine residues involved in glycation.** [The image was generated by PyMOL (0.99rc6 open-source) with the use of 1BMO.pdb].

*In vitro* or *in vivo* studies that have been performed on structural properties of glycated albumin include both human and bovine albumin. Comparing sequences of human and bovine albumin reveals a homology close to 80% [78]. Observed differences are mainly proposed to be structurally conservative, e.g. hydrophobic amino acids are replaced by other hydrophobic amino acids and therefore the main three dimensional structures is the same. The glycation of albumin induces several structural modifications, including an increase in total molecular weight of the protein due to the glycation [79]. Non-enzymatic glycosylation of albumin occurs at multiple residues such as arginine, lysine and also cysteine. So far, a lot of studies focused on the main sites modified by glycation for serum albumin have been done *in vivo*. Lysine, arginine and cysteine residues are subjected to glycation mostly because of their high nucleophile properties. Lysine-525 (Figure 2) is considered to be the predominant site of the of human serum albumin *in vivo* glycation which constitutes 30% of the overall glycation of the protein by glucose [80]. Alongside with Lys-525 which appears to be the most reactive glycation site, in native conformation, other lysine residues, such as 199, 276, 281,378, 439, and 545 have been found to have lower participation than Lys-525 in overall glycation [81,82]. For example, Lys-199 accounts for only 5% of total glycation. In addition, other residues of less importance have also been identified [80] which are located in the vicinity of known drug binding sites in HSA [82]. These charged amino acid residues at physiological pH may have an acid-based role for Amadori rearrangement. The Lys-199 and Lys-281 are close to disulfide bonds, which present a positively charged amino group, close to these sites and higher accessibility of some amine residues depends on the tertiary structure conformation of albumin.

Regardless of residues located at the amino terminal of the albumin, evidence for the reactiviy of other lysine residues, such as Lys-12, Lys-233, Lys-317, or Lys-351, is less certain [83]. This hypothesis is established by the fact that *in vivo* copper-albumin complex cannot be glycated. The extent of glycation depends on the glycemic status and also the half-life of the protein.

Other researches propose lysine-524 (equivalent to Lys-525 of HSA) as the major glycation site in BSA [84] and Lys-275 (equivalent to Lys-276 of HSA), Lys-232 (equivalent to Lys-233 of HSA) and Lys-396 are susceptible to be glycated [84].

Although less than lysine residues (24 for 59 lysine residues), arginine residues can also be involved in glycative modification of HSA (Figure 2). Arg-410 appears to be the predominant glycation site [85] and other involving but not major residues such as Arg-114, Arg-160, Arg-186, Arg-218 and Arg-428, have also been identified [82].

Finally, the thiol group of cysteine residues is powerful nucleophile, which can also be glycated *in vitro* by methylglyoxal to give rise to AGEs such as S-carboxymethyl-cysteine (CMC) [86]. CMC occurrence in plasma from diabetic patients, suggest the involvement of Cys-34 in the glycation process [87].

Glycoxidation of albumin is accompanied by structural modifications in both *in vitro* and *in vivo* glycation process. The interrelationship between glycation and amyloid formation is interesting in this regard, as criteria for glycation completion was the production of the amyloid nanofibril after 20 weeks of incubation with glucose. This has caused a decrease in hydrophobicity of the protein [88] due to the transition in albumin from a helical to a beta-sheet structure. Glycation induced aggregation is not necessarily associated with secondary structure modification. In addition, the glycation process could result in an overall stabilization in both tertiary and secondary structure of the protein which increases the protein stability and improves the protein life time [33].

### Biological impact

Glycated albumin has an important clinical implication, since it is involved in the damages associated with the diabetes mellitus, such as retinopathy, nephropathy, neuropathy and coronary artery disease [89]. In most studies, the deleterious effects of glycated albumin have been highlighted for instance the physiopathological correlation between glycated albumin and diabetic renal deficiency and microangiopathy [90] and also diabetic retinopathy [91]. Several *in vitro* studies have shown the implication of glycated albumin in platelet activation and aggregation [92]. Beside platelets, the lowering aggregation effects of glycated albumin have also been demonstrated in erythrocytes [93].

Another pathogenic implication of glycated albumin can also be observed in glucose metabolism of both skeletal muscle and adipocyte cells [94]. It has been found that in mouse, adipocyte cell lines, albumin-derived species triggers generation of intracellular ROS leading to an inhibition of glucose uptake which also cause attenuation of adipocyte insulin sensitivity and microangiopathy [90]. More recently, proteins such as nucleophosmin in monocyte like cell membrane and Calnexin, a transmembrane protein, were reported to serve as receptors for Amadori-glycated albumin [95].

### Glycated albumin, a possible specific marker for diabetes

The glycation process of proteins occurs in a higher amount in diabetic patients compared to non-diabetic individuals. This is strongly involved in the development and progression of chronic diabetic complications. The two main clinical parameters of diabetes are the glycated hemoglobin (HbA1C) and the blood glucose levels [96]. The first parameter is as long term indicator of diabetes, because of long term half life of erythrocytes(about 120 days) and could reflects the glycemic control state over the past 2 months, But the measurement of glucose is a short-term indicator and reflects diabetic status over a 24 h period. However, several studies have reported that in some case, HbA1C values should be considered cautiously. As a matter of fact, glycated hemoglobin levels have invalid correlation to blood glucose levels in patients with hemolytic anemia, or those having hemodialysis or iron deficiency [97].

Thus in numerous case such as hemolytic or renal anemia and liver cirrhosis, HbA1C gives incorrect values and is not suitable marker as a control [98]. Glycated albumin, because of its shorter half-life (21 days) compared with hemoglobin, could be used as a shorter-term glycemic control for diabetes. The glycated albumin level could not to be easily altered by abnormal hemoglobin metabolism [99]. This advantage of glycated albumin is based on two facts. First, the amount of *in vivo* non-enzymatic glycation of albumin is approximately 9 times more than HbA1C [80]. Secondly, albumin glycation reaction occurs ten times more quickly than hemoglobin glycation [80] so, the glycation phenomenon in plasmatic protein occurs more easily than hemoglobin, which all make the glycated albumin a good additional marker for evaluating glycemic control in type 1 and 2 diabetes [100].

In some studies glycated albumin is suggested as an alternative marker for glycemic control in many diabetes complications, including nephropathy [101], retinopathy [102] and Alzheimer’s disease [72] and also in the case of hemodialysis patients [103] or gestational diabetes [97]. All these data support the possibility use of glycated albumin in the detection of short-term changes in glycemic controls. Glycated albumin levels, determined in different *in vivo* studies for different pathologies associated to diabetes mellitus, could be consulted in [33].

It should be mentioned that in some cases such as thyroid dysfunction, nephrotic syndrome or liver cirrhosis which the amounts of albumin is affected, and so, glycated albumin level is not a suitable indicator [104]. Similarly, glycated albumin could be influenced by other conditions, such as body mass index (BMI) [105] or the age of diabetic patients [106]. Therefore a combined detection of HbA1C and glycated albumin may improve the efficacy of diagnosis and improvement of a novel therapeutic potential [107].

### *In vitro* models of glycated albumin

As highlighted in the previous sections, studies on the glycation process of Human Serum Albumin (HSA) is of special interest, due to its clinical significance. This final section intends to introduce *in vitro* models of albumin glycation, as a mean to study this phenomenon under controlled conditions. The significant impact of glycation on the protein structure could be observed more conveniently by *in vitro* techniques such as the use of heat and pressure [108]. HSA has approximately 58 lysine residues [80]. As mentioned previously, during the glycation process, different sugars and sugar phosphates could undergo traditional Maillard reactions leading formation of various products [109]. Syrový et al. glycated albumin (5 mg/ml) using Hepes (4-(2-hydroxyethyl)-1-piperazine ethane sulfonic acid) buffer and carbohydrates including glucose, fructose, galactose, ribose and glyceraldehydes for 20 days at 37°C. They showed that the incubation of albumin was occurring more efficiently in presence of ribose, glyceraldehydes and galactose respectively [110]. Another research was done by Schmitt et al. to study modifications taking place during glycation of 4 mM HSA in different glucose concentrations (5–500 mM) and showed that there is a linear relationship between these two parameters. They found that the percentage of reacted lysines was increasing rapidly up to 200 mM glucose [111]. GhoshMoulick et al. compared the glycation process of albumin and two other proteins, namely hemoglobin and lysozyme, and demonstrated that the ability of albumin glycation is positioned between two others [112]. Sattarahmady et al. compared the ability of glucose, fructose, and ribose in glycation of HSA and generation of amyloid structures and demonstrated that the prolonged exposure of HSA has a detergent-like effect on HSA structure and leads to partial unfolding and hydrophobic surfaces exposure, and finally amyloid formation [35]. In a previous study, they had monitored changes occurring in the three-dimensional structure of the protein, and the number of basic residues modified with glucose [88]. In order to study *in vitro* formation of AGE-HSA, protein concentration of 1–10 mg/ml have been used in phosphate buffer to be incubated with glucose, fructose, and ribose or other reducing sugars (generally at 500 mM) at 37°C in the dark [35,110,113,114]. After 2, 4, or 20 weeks of incubation, the glycation process could be observed as browning soluble materials. Recently, S. Barnaby et al. have made a comparison of modification sites formed on human serum albumin at various stages of glycation. It was found that the glycation pattern of HSA strongly depends on total amounts of glycation. Many modifications sites including K199, K281, and the N terminus, in addition to lysines 199 and 281, as well as arginine 428 were found in the tested samples. Lysine residues 93, 276, 286, 414, 439, and 524/525, as well as the N-terminus and arginines 98, 197, and 521, were also found to be modified at various degrees of HSA glycation [113].

Glycated HSA could forms micelle-like aggregates upon prolonged glycation. In these cases, glycation of HSA resulted in physicochemical changes including alterations in protein conformation, molecular weight, and *pI* and all these changes were in the direction of transition from a helical to a β-sheet structure and formation of nanofibrillar amyloid.

Although in all cases mentioned before, using glucose could be more compatible to what happens in the body, in some cases the glycation process is faster by the use of other reducing sugars, while some results could still be interpreted as relevant to the natural conditions. Similarly, using buffers more similar to the body conditions could present more reliable results for the glycation process.

## Conclusion

This review has outlined the glycation reaction of proteins including a discussion on the glycation process itself, and particularly with regard to albumin glycation. We give an overview of the role of glycation in the physiopathology of different diseases and specially highlight the properties of glycated albumin. The new aspect of glycated albumin being used as a disease marker, compared with HbA1C is also mentioned. Finally, an introduction to some *in vitro* models of glycated albumin is made. It is interesting to note the relationship found between *in vitro* glycation experiments and the propensity of proteins to form amyloid structures, a point that could be further explored as to its significance in hyperglycemic states.

## Competing interests

The authors declare to have no competing interests.

## Authors’ contributions

AA design of the study, SF helped to draft the manuscript, MH participated in the design of the study and overall manuscript architecture, AM conceived of the study. All authors read and approved the final manuscript.
